# Distinct Conformations, Aggregation and Cellular Internalization of Different Tau Strains

**DOI:** 10.3389/fncel.2019.00296

**Published:** 2019-07-09

**Authors:** Thomas K. Karikari, David A. Nagel, Alastair Grainger, Charlotte Clarke-Bland, James Crowe, Eric J. Hill, Kevin G. Moffat

**Affiliations:** ^1^School of Life Sciences, University of Warwick, Coventry, United Kingdom; ^2^Midlands Integrative Biosciences Training Partnership, University of Warwick, Coventry, United Kingdom; ^3^School of Life and Health Sciences, Aston University, Birmingham, United Kingdom

**Keywords:** tau protein, Alzheimer’s disease, frontotemporal dementia, MAPT mutations, oligomer, nuclear tau, cellular internalization, induced pluripotent stem cell-derived neurons

## Abstract

The inter-cellular propagation of tau aggregates in several neurodegenerative diseases involves, in part, recurring cycles of extracellular tau uptake, initiation of endogenous tau aggregation, and extracellular release of at least part of this protein complex. However, human brain tau extracts from diverse tauopathies exhibit variant or “strain” specificity in inducing inter-cellular propagation in both cell and animal models. It is unclear if these distinctive properties are affected by disease-specific differences in aggregated tau conformation and structure. We have used a combined structural and cell biological approach to study if two frontotemporal dementia (FTD)-associated pathologic mutations, V337M and N279K, affect the aggregation, conformation and cellular internalization of the tau four-repeat domain (K18) fragment. In both heparin-induced and native-state aggregation experiments, each FTD variant formed soluble and fibrillar aggregates with remarkable morphological and immunological distinctions from the wild type (WT) aggregates. Exogenously applied oligomers of the FTD tau-K18 variants (V337M and N279K) were significantly more efficiently taken up by SH-SY5Y neuroblastoma cells than WT tau-K18, suggesting mutation-induced changes in cellular internalization. However, shared internalization mechanisms were observed: endocytosed oligomers were distributed in the cytoplasm and nucleus of SH-SY5Y cells and the neurites and soma of human induced pluripotent stem cell-derived neurons, where they co-localized with endogenous tau and the nuclear protein nucleolin. Altogether, evidence of conformational and aggregation differences between WT and disease-mutated tau K18 is demonstrated, which may explain their distinct cellular internalization potencies. These findings may account for critical aspects of the molecular pathogenesis of tauopathies involving WT and mutated tau.

## Introduction

Tauopathies are characterized by the intracellular accumulation of NFTs composed of aggregated misfolded tau (Arriagada et al., 1992; Spillantini and Goedert, 2013). NFTs can be formed when tau proteins lose affinity for MTs and bind to themselves, forming insoluble aggregates that can subsequently develop into NFTs (Goedert, 2016). Although monomeric tau – the physiological form of the protein – is natively unfolded, it becomes progressively enriched in β-sheets through initial aggregation into soluble conformers and then into PHFs and NFTs (Barghorn et al., 2004; von Bergen et al., 2005). Although growing evidence indicates that tau dysfunction can cause neurodegeneration, the mechanisms involved are not well understood (Guo and Lee, 2014; Walsh and Selkoe, 2016). Many recent studies have shown that the typical temporal and spatial accumulation of tau inclusions in specific brain regions in AD and other tauopathies may be due to the propagation of aggregated tau between synaptically connected neurons (Frost et al., 2009; Guo and Lee, 2014; Usenovic et al., 2015). This inter-neuronal propagation of abnormal tau, often referred to as “spread,” is understood to initiate the structural and functional differences observed in affected brain regions (Boluda et al., 2015; Usenovic et al., 2015). As a predominantly cytosolic protein, the spread of tau is expected to involve repeated cycles of the following steps: (i) secretion of tau, either in aggregated or soluble forms, from donor neurons; (ii) uptake of extracellular tau into specific neighboring neurons; (iii) induction of endogenous tau aggregation in the recipient neurons; and (iv) the secretion of at least part of this internalized tau-endogenous tau protein complex (Clavaguera et al., 2009; Frost et al., 2009; Guo and Lee, 2011; Lasagna-Reeves et al., 2012a; Michel et al., 2014). Nonetheless, the molecular mechanism governing this process is poorly understood (Walsh and Selkoe, 2016). As such, an improved understanding of the pathways involved in these diseases could lead to the development of new therapies.

**FIGURE 1:**
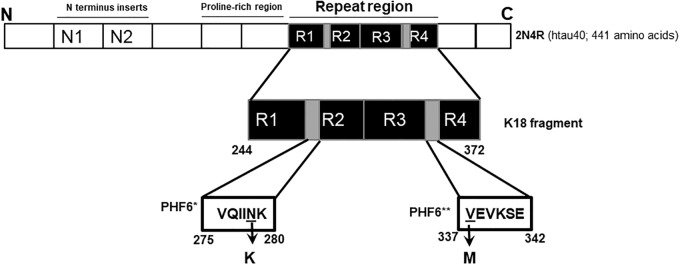
Schematic illustration of the N279K and V337M mutations in tau. The K18 fragment used in this study was constructed based on the full-length human brain tau isoform (2N4R; htau40). The N279K mutation is located in the alternatively spliced second repeat region (R2), specifically within the hexapeptide motif at the start of R2 (PHF6^∗^; gray). The V337M mutation can be found at the start of the fourth repeat domain, and falls within the third hexapeptide motif (PHF6^∗∗^; second gray region).

The efficiency of the inter-cellular propagation of pathological tau appears to be influenced by the conformations of both the seed (internalized) and the seeded (recipient endogenous) proteins (Goedert et al., 2014; Sanders et al., 2014; Wegmann et al., 2015; Alonso et al., 2016; Kaufman et al., 2016). This has therefore led to the *tau strain hypothesis*, which proposes that different aggregated tau conformers (distinct strains) will have distinct pathology-initiating capacities because they interact with endogenous tau differently (Sanders et al., 2014; Gerson et al., 2016). This hypothesis is supported by several studies (Clavaguera et al., 2013; Sanders et al., 2014; Boluda et al., 2015; Kaufman et al., 2016; Narasimhan et al., 2017; Strang et al., 2017). For example, intracerebral introduction of human tauopathy brain isolates into brains of the non-filament-forming ALZ17 mice led to distinctions in the induction of tau inclusions, dependent on the source of homogenate (Clavaguera et al., 2013). Inclusions formed after injecting isolates from human AD, progressive supranuclear palsy, argyrophilic grain disease, CBD or tangle-only dementia propagated between neighboring brain regions. Conversely, propagation was not observed for aggregates formed in the case of Pick’s disease. Importantly, these results were similar to those recorded after seeding WT mice with the same isolates, suggesting that the genetic background of the animals did not influence the outcome (Clavaguera et al., 2013). Similarly, intracerebral introduction of brain extracts from human CBD and AD cases into young tangle-forming PS19 mice brains resulted in the formation and propagation of tau inclusions and neuronal loss that appeared specific to selected cell types (Boluda et al., 2015). Extracts from CBD patients led to tau inclusions limited to oligodendrocytes of the fimbria and white matter around the injection site, while tau aggregates formed after introducing AD extracts were found in neuronal perikaya without obvious oligodendrocyte participation (Boluda et al., 2015). These results suggest that tau isolates from different sources can exhibit distinctions in their ability to induce tau inclusion formation in the recipient cell type, which may be due to conformational differences between the endogenous tau and the initiating tau seeds. Recently, both cell-type specificity and differential endogenous tau recruitment have been implicated as determinants of inter-cellular propagation of human tauopathy brain extracts (Narasimhan et al., 2017). Furthermore, recombinant full-length and truncated forms of tau, such as the four-repeat K18 region comprising residues 244–372 of full-length tau 441 (Figure 1), have been shown to initiate propagation by inducing endogenous tau aggregation (Guo and Lee, 2011; Iba et al., 2013; Michel et al., 2014; Falcon et al., 2015; Usenovic et al., 2015). This seeding capacity may be due to conformational similarities between some forms of recombinant tau applied as seeds and the endogenous tau, because these recombinant forms can take on endogenous tau conformation in some cell models (Falcon et al., 2015).

Whilst there is mounting data supporting the inter-cellular propagation of aggregated recombinant WT tau, we know little about FTD (Bodea et al., 2016). Many familial FTD cases are characterized by single point mutations in the *MAPT* gene, which encodes the tau protein. Specific FTD-associated *MAPT* mutations have been shown to alter the aggregation, β-sheet propensity, isoform ratio and MT binding properties of the WT tau (Hong et al., 1998; Barghorn et al., 2000; von Bergen et al., 2001; Combs and Gamblin, 2012; Kaniyappan et al., 2017). It is, however, unclear if aggregated tau proteins from FTD with *MAPT* mutations are conformationally distinct from WT tau, and if they have different cellular internalization behavior and co-localization dynamics with endogenous tau. Strang et al. (2017) recently demonstrated that the ability of exogenous WT tau K18 fibrils to induce aggregation in cells over-expressing tau carrying different FTD mutations is uniquely dependent on the specific mutation and its surrounding amino acid sequences. However, several questions remain, such as what structural features determine these distinct activities? Do the conformation and aggregation characteristics of tau variants influence their cellular internalization and endogenous tau interaction? Furthermore, it has been shown that the N279K variant of the tau 250–300 fragment localizes to the nucleus more efficiently that the WT when overexpressed in a mammalian cell line (Ritter et al., 2018). It is yet to be shown if exogenously applied aggregates of this and other FTD variants of tau can enter recipient cells and localize to the nucleus, in a process suggestive of cell-to-cell propagation. Answering these questions is critical to understanding the mechanisms by which AD and non-AD tauopathies develop, and will help to establish if selectivity is required in drug development for treating tauopathies.

In this study, we investigated the *in vitro* conformation and aggregation of tau K18-WT and two FTD variants, namely V337M and N279K. The N279K mutation is a part of the PHF6^∗^ hexapeptide motif (^275^VQIINK^280^) but the V337M mutation is located in the PHF6^∗∗^ hexapeptide motif (^337^VEVKSE^342^)(Li and Lee, 2006; Grüning et al., 2014). Since these motifs critically regulate tau aggregation and seeding behavior, amino acid mutations were hypothesized to alter these processes (von Bergen et al., 2000, 2001; Li and Lee, 2006; Grüning et al., 2014; Falcon et al., 2015). In addition, the N279K and V337M are located in the second and the fourth MT binding repeat regions, respectively (Figure 1). Thus, the characterization of these mutations should offer new understanding into the consequences of disease-related alterations in alternatively- and constitutively spliced domains. The mutations also have distinct pathological consequences in human brains (Hong et al., 1998), suggesting differential effects on tau aggregation and structure.

We have shown that the structural morphology and immuno-reactivity of K18-WT aggregation products were distinctively altered in the presence of each mutation. Furthermore, internalization of exogenous structurally defined oligomers into SH-SY5Y cells was significantly increased in the presence of each FTD mutation. However, the sub-cellular localization and endogenous tau co-localization patterns were similar between the three protein variants, both in SH-SY5Y cells and differentiated human induced pluripotent stem cell (hiPSC)-derived neurons. This may suggest shared mechanisms of cellular uptake, but differences in the efficiency of uptake, between WT tau K18 and its V337M and N279K FTD variants. Together, the results give new insights into the structural and functional dynamics of WT and FTD tau. We hypothesize that the distinct conformation and aggregation behaviors of the tau K18 variants may influence their inter-cellular propagation characteristics. These findings may help to explain important aspects of similarities and heterogeneity in tauopathies involving WT and mutated tau.

## Materials and Methods

### Materials

Heparin (#H3393), SH-SY5Y cells (#94030304), sodium chloride (#S/3160/60), sodium phosphate dibasic (#S9763-500 G), sodium phosphate monobasic dihydrate (#04269-1 KG), Triton X-100 (#X100-500 ML), Tween (#P9416-100 ML), F12 Ham (#51651C), L-Glutamine (#G7513), fetal bovine serum (#F2442) and antibiotic antimycotic acid (#A5955) were purchased from Sigma Aldrich, St. Louis, Missouri, United States. X1 Amersham Protran 0.45 μm electrochemiluminescence nitrocellulose membrane (#15259794) and Amersham electrochemiluminescence system (#RPN2108) were obtained from GE Healthcare, Buckinghamshire, United Kingdom. EDTA (#D/0700/53) was bought from Fisher Scientific, Loughborough, United Kingdom. PIPES (#A16090) was bought from Alfa Aesar, Heysham, United Kingdom, whilst DMSO (cell culture-grade; #P60-36720100) was from PAN-Biotech GmbH, Aidenbach, Germany. CL-XPosure Film (#34089) and ProLong Gold antifade mounting medium (#P36934) were purchased from Thermo Fisher Scientific, Rockford, Illinois, United States. CellMask^^®^^ Deep Red plasma membrane stain (#C10046), Hoechst 33342 (#H21492) and Alexa Fluor^^®^^ 488 C5-maleimide (#A10254) were bought from Molecular Probes, Eugene, Oregon, United States. Lactate dehydrogenase (LDH) cytotoxicity assay kit (#88954) was obtained from Pierce Biotechnology, Rockford, Illinois, United States. TCEP (#A2233,0001) was procured from Applichem GmbH, Damstadt, Germany. SynaptoRed^TM^ C2 (FM4-64; #70021) was obtained from Biotium Hayward, California, United States. CellView^TM^ dishes (#627975) were procured from Greiner Bio-One, Kremsmünster, Austria. Formvar/carbon-coated 300-mesh copper grids (#S162) and mica (#G250-3) were purchased from Agar Scientific, Stansted, Essex, United Kingdom. Neural precursor cells (#ax0016), Axol neural maintenance medium kit (#ax0031), and Surebond reagent (#ax0041) were purchased from Axol Bioscience, Little Chesterford, Cambridge, United Kingdom. Marvel dried skimmed milk was obtained from Premier Foods Company, United Kingdom. Antibodies used have been described in Table 1.

**Table 1:** Antibodies used in this study.

Antibody	Epitope	Host species	Vendor	Dilution
Primary antibodies		
Polyclonal rabbit anti- human tau (#A0024)	The MT binding repeat of tau	Rabbit	Dako	1:1000
T22 oligomeric tau (#ABN454)	Tau MT binding domain	Rabbit	Merck	1:1000
HT7 anti-human tau (#MN1000)	Amino acid sequence ^159^PPGQK^163^ of full length human tau 2N4R	Mouse	Thermo Scientific	1:100
Alexa Fluor^^®^^ 647-conjugated anti-nucleolin (#ab198580)	Human nucleolin	Mouse	Abcam	1:500
Secondary antibodies		
Goat anti-rabbit IgG (#31460)	–	Goat	Thermo Fisher Scientific	1:1000
Alexa Fluor^^®^^ 594-conjugated AffiniPure goat anti-mouse	–	Goat	Jackson ImmunoResearch	1:100

### Site Directed Mutagenesis, Protein Expression and Purification

Detailed description of the construction of the pProEx-HTa-Myc-K18 plasmid, and sequence-verified site-directed mutagenesis to create plasmid variants encoding the V337M and N279K mutations has been provided previously (Karikari et al., 2017). To prevent potential adverse functional effects of maleimide labeling, the native cysteines located in the central core of the MT binding region were each changed to alanine (C291A, C322A) and a new cysteine placed outside the central core of this region (I260C). These cysteine modifications were achieved by site-directed mutagenesis as described (Karikari et al., 2017). Several studies have used this approach, understood not to impact tau aggregation behavior, to study the biochemical properties and internalization of extracellular tau (Kumar et al., 2014; Michel et al., 2014; Shammas et al., 2015). The same cysteine-modified proteins were used in all experiments. Plasmids encoding N-terminal 6xHis-c-Myc K18-WT, K18-V337M and K18-N279K were expressed in BL21 (DE3)^∗^pRosetta *Escherichia coli*, purified with immobilized metal affinity chromatography and quantified with the Bicinchoninic acid assay as described previously (Karikari et al., 2017).

### Filament Assembly and Structural Characterization Using Negative-Stain Transmission Electron Microscopy (TEM)

K18-WT or its variants (38.5 μM) mixed with 125 μM heparin were incubated at 37°C without agitation for 4 or 45 days. Sodium phosphate buffer pH 7.4 was the diluent buffer used in all biochemical experiments unless otherwise indicated. All incubation was performed using an Eppendorf Mastercycler thermocycler, and aliquots removed for TEM. Five microliters of the indicated proteins were pipetted onto glow-discharged formvar/carbon-coated 300-mesh grids for 2 min, and filter paper strips used to remove unbound samples. Thereafter, 5 μl of 2% uranyl acetate was added for 2 min, and the excess removed as previously before imaging. Quantitative analyses of filament lengths and widths were performed using Image J.

### Dot Blot Analysis of Protein Conformation

Protein variants at 55 μM were incubated at 37°C to promote aggregation. Aliquots removed at the given times were snap-frozen and stored at -80°C until needed. After diluting samples 1:2, 2 μl of the diluted samples were spotted onto 0.45-μm nitrocellulose membrane, air-dried, blocked for 30 min at room temperature with 10% non-fat milk in phosphate buffered saline (PBS), and washed five times using 10% Tris buffered saline-0.05% Tween. The membrane was incubated for 2 h in anti-tau #A0024 or T22, washed as previously, and incubated with anti-rabbit immunoglobulin G (Table 1) for a further 2 h. Each antibody was diluted 1:1000. The membrane was then washed five times, developed using electrochemiluminescence reagents (Amersham), imaged and analyzed as previously described (Karikari et al., 2017).

### Preparation and Characterization of Labeled Oligomers

Proteins were treated with five times excess of TCEP for 1 h, and then treated with 4× Alexa Fluor 488-C5-maleimide in the presence of 1× BRB buffer (1 mM KCl, 80 mM PIPES, and 1 mM EDTA pH 6.8). Free fluorophore was dialyzed out against dialysis buffer (50 mM Tris HCl pH 7.5, 100 mM NaCl) in Slide-A-Lyzer MINI Dialysis Device (5 K MWCO; Thermo Fisher Scientific) with punctured buffer reservoirs placed in containers that can hold 2 L of dialysis buffer. The buffer was changed every 2 h with a total of five buffer replacements. Labeling was confirmed using non-denaturing sodium dodecyl sulfate-polyacrylamide gel electrophoresis followed by ultraviolet light exposure. The efficiency of labeling was routinely estimated using Beer’s law and the molar extinction coefficient of 72,000/cm/M for Alexa Fluor 488-C5-maleimide.

### SH-SY5Y Cell Culture

SH-SY5Y cells were cultured on 1:1 minimal essential medium/F12 Ham medium supplemented with 1% L-Glutamine, 15% fetal bovine serum, and 1% antibiotic antimycotic acid (10,000 units penicillin, 10 mg streptomycin and 25 μg amphotericin B). All experiments were performed using cells between passages two and ten. Cells were grown at a density of 200,000 cells/ml in CellView^TM^ dishes, 10 μM labeled tau oligomers added to the medium, and incubated for 24 h at 37°C, 5% CO_2_. After removing the used medium, the cells were washed with warm PBS and fresh medium with 2 μM Hoechst and 1:1000 dilution of CellMask Deep Red added before imaging.

### hiPSC-Derived Neuronal Culture

Neural precursors – from cord blood CD34+ cells of a newborn female – that had been cultured for 21 days from iPSCs to neural precursor cells were bought from Axol Bioscience. Grade 12-well tissue culture plates (Corning, New York, United States) or plates containing plasma-cleaned and ethanol-sterilized 13 mm glass coverslips (Menzer, Thermo Fisher Scientific) were each pre-coated with 250 μl/cm^2^ ReadySet reagent (Axol Bioscience) and kept at 37°C, 5% CO_2_ for 45 min. The plates were then rinsed 4× with double-distilled water. Thereafter, Surebond reagent (Axol Bioscience) diluted in DPBS was added to the plates at 200 μl/cm^2^ and kept at 37°C, 5% CO_2_ for at least 1 h. Cells were seeded on plasma-cleaned 13 mm glass coverslips at 25,000 cells/cm^2^ final density and stored at 37°C, 5% CO_2_. The media was changed every other day for 14–16 days using the Axol Neural Maintenance Medium kit (#ax0031, Axol Bioscience). Phase contrast microscopy images taken periodically with the EVOS XL Core Imaging System (Life Technologies) and the expression of neuron-specific markers (data not shown) were used to monitor neuronal differentiation. Tau K18 oligomers diluted to 5 μM in neural maintenance medium were applied to cultured neurons and kept at 37°C, 5% CO_2_ for 24 h, after which the used medium was carefully removed and the neurons washed with DPBS before the addition of fresh medium.

### Immunocytochemistry

SH-SY5Y cells: the appropriate antibodies or dye were applied to the cells as described above and images taken with confocal microscopy without fixing. In internalization analysis, fresh media containing FM4-64 (2 μM) and Hoechst was added to cells after PBS wash as explained above. The cells were imaged after 30 min incubation at 37°C and 5% CO_2_. Nucleolin co-localization was investigated by adding a 500× dilution of an Alexa Fluor-647-labeled anti-human nucleolin (Table 1) in tau-free medium to tau oligomer-treated cells after PBS wash as described above. Cells were imaged after 2 h incubation at 37°C and 5% CO_2_. Cells were imaged without fixing.

hiPSC-derived neurons: Neurons were fixed with 4% paraformaldehyde for 30 min, twice washed with DPBS and then cleaned with permeabilizing solution (0.2% Triton in DPBS). Thereafter, the fixed neurons were stored in blocking buffer (DPBS containing 0.2% Triton and 2% BSA) for 1 h. Subsequently the cells were incubated with primary antibody and Hoechst for 1 h at 37°C and 5% CO_2_. The fixed neurons were washed thrice, each for 5 min, with blocking buffer to remove unbound primary antibody and then incubated for 1 h with secondary antibody diluted in blocking buffer. After incubation with the secondary antibody, the wash steps were repeated. The prepared coverslips were rinsed with distilled water and slide-mounted with ProLong Gold antifade mounting medium. Slides were incubated in the dark for at least 24 h before imaging. Details of antibodies used are available in Table 1.

### Confocal Microscopy

An LSM 710 (Leica) equipped with an C-Apochromat 63×/1.20 W Korr M27 objective lens was used to image SH-SY5Y cells. hiPSC-derived cortical neurons were imaged with the 63× objective of a Leica STP 6000 microscope. Images were analyzed with Image J. Internalization data are expressed as integrated density (area × mean fluorescent intensity). Identical imaging settings were used for each set of experiments (at least *n* = 3).

### LDH Assay

The Pierce LDH cytotoxicity assay kit was used. SH-SY5Y cells were seeded at 20,000 cells/well in Falcon 96-well plates and the indicated oligomer concentrations added. Controls included spontaneous cytotoxicity (cells + 10 μl sterile water), media only (no cells) and maximum control (membrane lysed with a manufacturer-supplied reagent). Cells were incubated for 72 h at 37°C 5% CO_2_. After incubation, lysis buffer was added to the maximum control wells and the plate incubated for a further 45 min. An aliquot (50 μl) of the sample mix in each well was transferred to a new 96-well plate and 50 μl reaction mix from the kit added to each well before incubation in the dark for 30 min. Stop solution (50 μl) was added and absorbance measured at 490 and 680 nm. Background readings at 680 nm were subtracted from corresponding 490 nm readings and LDH release (cytotoxicity) for each condition calculated by dividing that condition’s LDH activity value by the value for the maximum LDH activity control, and subtracting the spontaneous LDH activity value from both the numerator and the denominator.

### Statistics

Statistical analysis was done using Prism 6 (GraphPad Inc., CA, United States) at 95% confidence interval. The D’Agostino and Pearson test was used to test the datasets for normality. Whenever appropriate, parametric analysis was performed using one-way ANOVA with Tukey’s *post hoc* test. Conversely, non-parametric datasets were analyzed using Kruskal–Wallis test with Dunn’s *post hoc* test. Data are shown as mean ± standard error of the mean.

## Results

The K18 fragment consists of residues critical for tau aggregation and conformation, including the hexapeptide motifs at the beginning of repeat domains two (^275^VQIINK^280^), three (^306^VQIVYK^311^), and four (^337^VEVKSE^342^) (Figure 1). These motifs control aggregation by modulating tau-tau interactions that lead to conformational changes from random coil to cross β-sheet structures (von Bergen et al., 2000, 2005; Li and Lee, 2006; Grüning et al., 2014). Moreover, protomers comprising residues 306–378 form the core of tau filaments, with their differential packing mechanisms determining the type of aggregates formed (Fitzpatrick et al., 2017). Furthermore, the protein’s aggregation-seeding competence, partly responsible for its inter-cellular propagation, is held in the repeat region and its fragments consisting of specific motifs (Guo and Lee, 2011; Michel et al., 2014; Stöhr et al., 2017). As the repeat region contains residues that control tau aggregation, conformation and propagation, the K18 fragment was used to study potential effects of specific FTD mutations on these properties.

### Familial FTD Mutations Alter the Ultra-Structure of Tau K18 Aggregates

**FIGURE 2:**
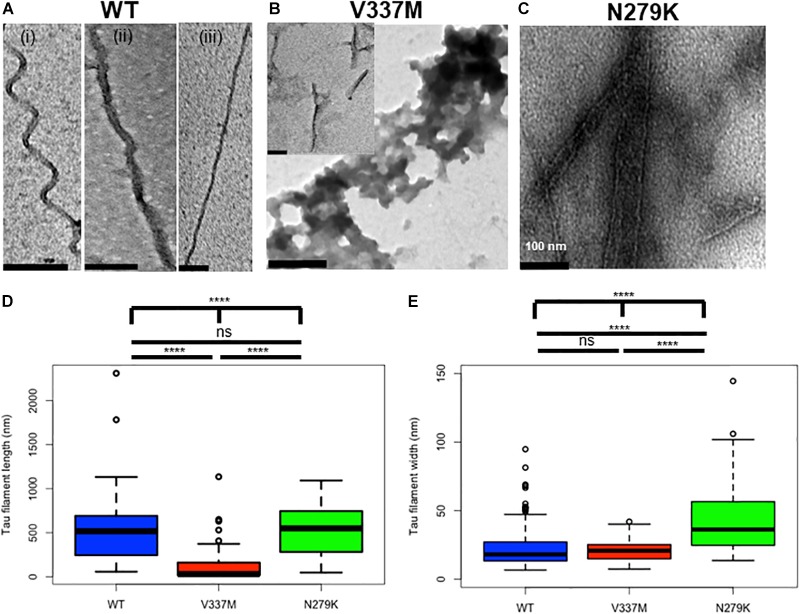
Structural changes in tau K18 filaments formed in the presence of the V337M and N279K FTD mutations after 4 days of incubation. **(A)** Representative electron micrographs of K18-WT, showing PHFs and SFs. **(B)** Representative electron micrographs of K18-V337M, which formed amorphous aggregates and short filaments (examples shown in the main figure and inset, respectively). **(C)** Representative electron micrographs of K18-N279K, showing bundled filaments of different shapes and widths. Scale bar = 100 nm for all images. Extended examples of the tau aggregates shown here can be found in Supplementary Figure S1. **(D,E)** Box plots of filament lengths and widths, respectively for the tau K18 variants. The plots show the results of a total of 73, 112, and 47 analyzed fibrillar structures for K18-WT, K18-V337M, and K18-N279K, respectively across *n* = 3 independent experiments using different batches of purified proteins. Kruskal–Wallis test followed by Dunn’s *post hoc* test; ^∗∗∗∗^ = *p* < 0.0001, ns, not significant.

Negative-stain TEM was used to study effects of the N279K and V337M mutations on tau K18 aggregation. Each sample was incubated with heparin at 37°C for four days (96 h) before TEM analysis. After this aggregation period, K18-WT mostly formed PHFs and, to a lesser degree, SFs. Both phenotypes of K18-WT have been reported previously (Barghorn and Mandelkow, 2002). The PHFs had different degrees of twisting, which increased up to ∼45° (Figure 2A and Supplementary Figure S1A). Unlike the PHFs, the SFs had no obvious helical structures (Figure 2Aiii). Tau K18-V337M aggregates had two types of phenotypes: large amorphous aggregates (Figure 2B main image) and short filamentous aggregates (Figure 2B inset). On the other hand, K18-N279K formed thick bundles of filaments of various width and length (Figure 2C). Supplementary Figure S1 is an extension of Figure 2, showing representative images from *n* = 3 independent experiments using different batches of purified proteins which further demonstrate the variety of aggregates formed by each tau K18 variant.

Filament length was significantly different when comparing all three proteins (*p* < 0.0001, Kruskal–Wallis test; mean lengths = 510.8 ± 42.3 nm, 106.6 ± 15.4 nm, and 534.8 ± 42.9 nm for WT, V337M, and N279K K18, respectively). Note that for K18-V337M, only the fibrils, but not the irregular amorphous structures, were analyzed. Filaments from both K18-WT and K18-N279K were significantly longer than those formed by K18-V337M (*p* < 0.0001, Kruskal–Wallis test with Dunn’s *post hoc* test; Figure 2D). Filament length between K18-WT and K18-N279K were not significantly different to each other (*p* > 0.9999, Kruskal–Wallis test with Dunn’s *post hoc* test). However, the three protein variants had significantly different filament widths (*p* < 0.0001, Kruskal–Wallis test; mean filament width = 27.9 ± 4.2 nm for K18-WT, 21.1 ± 0.8 nm for K18-V337M, and 53.0 ± 8.3 nm for K18-N279K), with K18-WT and K18-V337M filaments being similar (*p* = 0.6698, Kruskal–Wallis test with Dunn’s *post hoc* test; Figure 2E). K18-N279K filaments were significantly wider than those of K18-WT and K18-V337M (*p* < 0.0001 in both cases, Kruskal–Wallis test with Dunn’s *post hoc* test; Figure 2E). Together, the morphology and structural properties of K18-WT aggregates significantly changed in the presence of the FTD mutations, causing a shift from PHFs and SFs to unstructured, amorphous aggregates and shorter but wider filaments for K18-V337M, with K18-N279K aggregating into bundled filaments of similar length to those of K18-WT but of increased width.

### Phenotypes of Tau K18 Aggregates Do Not Change With Further Incubation

**FIGURE 3:**
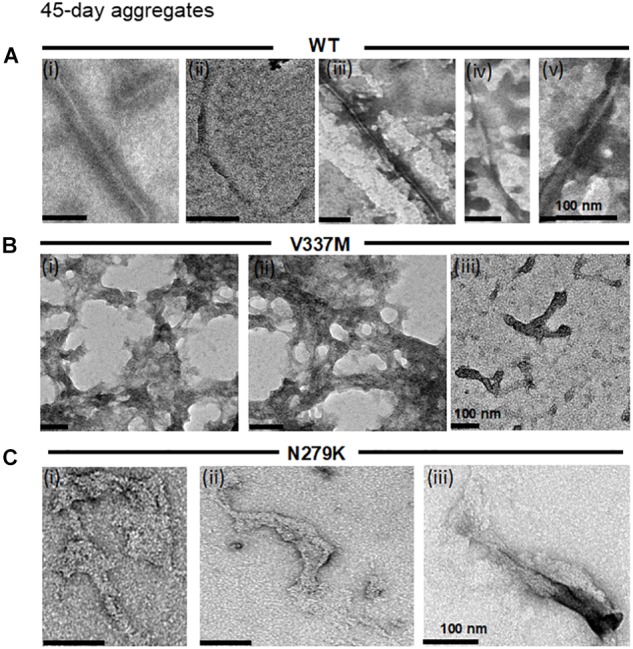
The distinct structural phenotypes of tau K18 aggregates observed in the presence of the FTD mutations do not change with extended aggregation reactions. **(A)** K18-WT formed PHFs and SFs, similar to those recorded in the 4 days assay in Figure 2A. **(B)** K18-V337M formed amorphous aggregates in addition to short filaments, similar to those observed in the 4 days aggregation assay in Figure 2B. **(C)** K18-N279K formed thick filament bundles whose structural features shares major similarities with those in Figure 2C. Scale bar = 100 nm for all images. Three independent experiments each performed with a different batch of purified protein.

As aggregation and fracture are thought to modulate the aggregation of tau K18 (Meyer et al., 2016). incubation times were increased to ascertain if the structural properties identified after 4 days would be maintained following prolonged incubation. Because aggregate conformation is partly determined by soluble tau recruitment onto template conformers (Margittai and Langen, 2004; Guo and Lee, 2014), it is conceivable that multiple cycles of aggregation and fracture may favor the growth of minor conformers which would otherwise be undetected in shorter experiments (Meyer et al., 2014, 2016). The previously described TEM experimental setup was employed to image samples obtained from extended incubation periods (45 days). The aggregates formed had the same morphological phenotypes as those seen after 4 days for all protein variants (Figure 3), indicating that the conformation of K18-WT aggregates is changed by the N279K and V337M mutations, and that these conformations persist during extended incubation.

### FTD Mutations Alter Immuno-Reactivity and Aggregation Products of Tau K18

The unique aggregation and structural properties of the FTD variants of K18 (Figure 2–3) are consistent with the hypothesis that they alter K18-WT conformation. In these experiments, heparin was added to accelerate the aggregation and filament formation process. However, the transient nature of heparin-treated tau aggregation can preclude the detection of important conformational changes. The tau proteins’ propensity to self-aggregate into filaments in the heparin-free state and the conformations they adopt during this process were therefore, studied.

**FIGURE 4:**
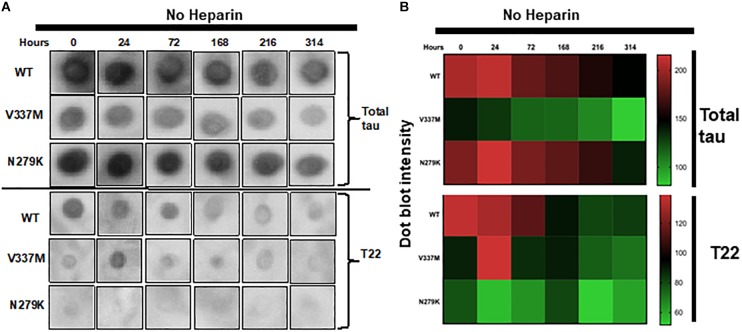
The FTD mutations change the immuno-reactivity of tau K18. **(A)** Dot blots of K18-WT and its FTD variants aggregating in heparin-free conditions probed with two different tau antibodies; total tau (A0024 from Dako) and the oligomer-preferring T22. The dot blot data shown here were from identical experiments, with uncropped images shown in Supplementary Figure S2. **(B)** Semi-quantification of dot blot intensity using heat maps.

Reactivity to specific antibodies (total tau A0024 (K9JA) and T22 oligomer conformation-preferring) was used to test for any further indication of distinct conformations, since proteinopathic protein variants with different conformations can lead have differential immuno-reactivity profiles (Hatami et al., 2017). Incubation time was extended to 314 h without agitation to allow PHF formation. Incipient immuno-reactivity was analyzed by dot blotting (Figure 4 and Supplementary Figure S2). K18-WT had the strongest immuno-reactivity to the total tau antibody A0024 at 0 h but this decreased over time (Figure 4A,B, upper panels). Similar immuno-reactivity, to A0024, of K18-N279K to that of K18-WT was observed, with the highest intensities recorded at early time points. However, K18-V337M had the lowest immuno-reactivity at each time point: the intensity at 0 h, the strongest recorded, was similar to the lowest intensity for K18-WT at 314 h (Figure 4A,B).

To gain insight into possible differences in oligomer conformation, reactivity to the oligomer-preferring T22 antibody was analyzed. Similar to its reactivity to A0024, K18-WT had its highest T22 immuno-reactivity at 0 h, which dropped sharply from 168 h onward (Figure 4A,B; lower panels). The reactivity of K18-V337M was low at all time points except at 24 h. K18-N279K had the least intensity to T22 at each time point (Figure 4A,B; lower panels and Supplementary Figure S2). This result implies that each protein variant folds differently with distinct epitope exposure during aggregation.

### Dominant Aggregate Conformers of the Tau K18 Variants Are Unaffected by Seeded Aggregation

Mutations in tau K18 can cause preferential amplification of specific aggregate conformers (Meyer et al., 2014). However, it is unknown if the dominant tau K18 conformers are affected by seeded aggregation. We therefore, studied if the mutation-induced dominant conformations of the tau K18 variants are affected by the addition of aggregated seeds from different tau K18 variants. Using the same experimental setup as in Figure 2, we seeded monomers of tau K18 WT, V337M, and N279K each with 10% volume of 4 days preformed aggregates from each protein variant. Negative-stain TEM imaging showed that seeded reactions (Supplementary Figures S3B–D) resulted in aggregate structures with the same phenotypes as those in seed-free experiments (Supplementary Figure S3A, same as in Figure 2) irrespective of the seed identity. These results suggest that the conformations of the tau K18 aggregates are not only be distinct but may also be incompatible, and that the majority species preferentially aggregate even when seeded with other conformers.

### Preparation and Characterization of Fluorescent K18 Oligomers for Functional Studies

Distinct structures of tau strains are thought to explain their divergent functional effects (Clavaguera et al., 2013; Boluda et al., 2015; Falcon et al., 2015; Narasimhan et al., 2017). We therefore, sought to take advantage of the conformational and aggregation differences of the tau K18 variants to investigate their possible influences on the cellular internalization of exogenously applied oligomers. To achieve this, Alexa Fluor-488-C5-maleimide-labeled oligomers were first prepared as detailed in section “Materials and Methods” and elsewhere (Karikari et al., 2019) before being used in *in vitro* cell culture assays. These stable oligomers were granular and non-fibrillar (Supplementary Figure S4).

### FTD Mutations Enhance Tau K18 Oligomer Internalization in SH-SY5Y Cells

Increasing evidence suggests that the cellular internalization of tau oligomers is an initial trigger of tau pathology (Usenovic et al., 2015; Kolarova et al., 2017). It is, however, not known if FTD with tau mutations are characterized by the same or similar mechanism. To address this, fluorescently labeled oligomers of K18-WT, K18-V337M, and K18-N279K were applied exogenously to SH-SY5Y cells and their internalization dynamics investigated.

**FIGURE 5:**
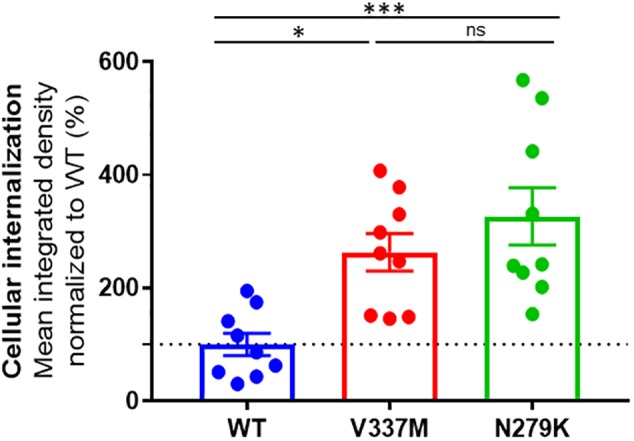
Distinct internalization of K18-WT, K18-V337M, and K18-N279K oligomers in SH-SY5Y neuroblastoma cells. Plot of tau K18 oligomer internalization in SH-SY5Y cells. For each condition, the same number of cells was treated with 10 μM of each protein oligomers as explained in section “Materials and Methods.” The graph shows % mean integrated density data normalized to WT. Internalization was significantly different between the tau K18 protein variants (*p* = 0.0007); significantly higher for both K18-V337M and K18-N279K against K18-WT (*p* = 0.0122 and *p* = 0.0006, respectively), using one-way ANOVA followed by Tukey’s *post hoc* test. The dotted line shows the mean internalization data for the WT (100%) to which the rest of the data was compared. ^∗^Signs refer to significance levels as used in the figure.

All three tau protein variants were internalized by SH-SY5Y cells (Supplementary Figures S5–S8). To account for both diffused and punctate phenotypes, we used integrated density (mean intensity x area occupied) to quantify internalized tau in experiments, where equal numbers of cells were seeded and treated with equimolar concentrations of each protein oligomers. The internalization data for each tau K18 variant was analyzed by normalizing the mean integrated density from representative images to the overall mean of the WT (Figure 5). Internalization was significantly different between the three protein variants (one-way ANOVA, *p* = 0.0007, F statistic = 10.08). The Tukey’s multiple comparison *post hoc* test showed that internalization was significantly higher for both K18-V337M (*p* = 0.0122) and K18-N279K (*p* = 0.0006) against K18-WT but not significantly different between K18-V337M and K18-N279K (*p* = 0.4524, Figure 5).

### Exogenous Tau K18 Oligomers Are Internalized by Endocytosis

**FIGURE 6:**
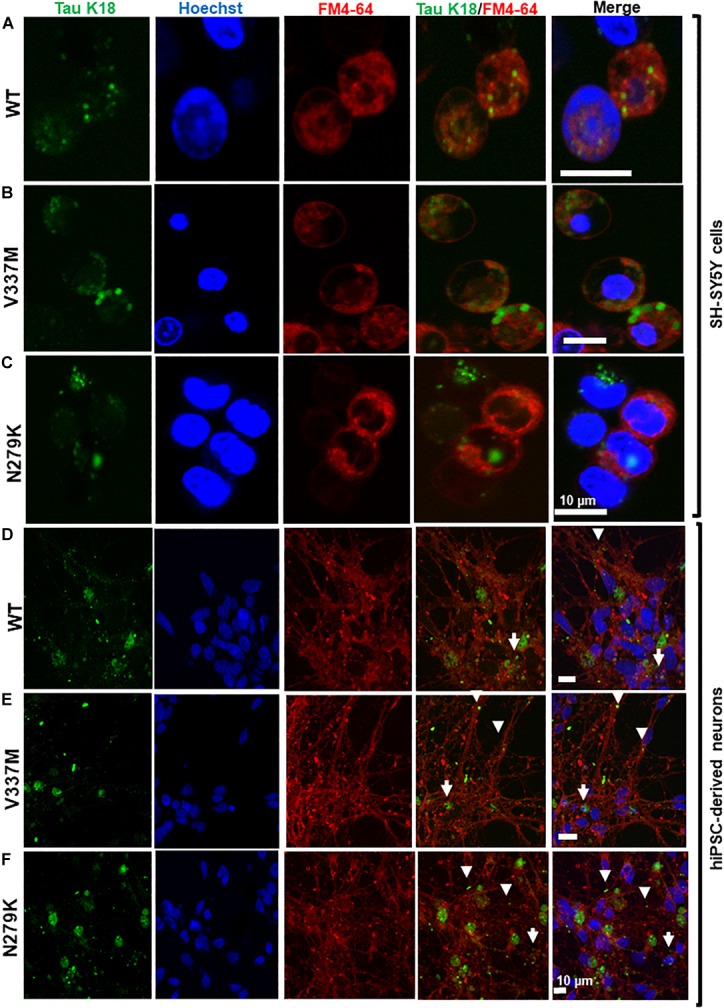
Extracellular tau K18 oligomers are taken up by endocytosis, with the internalized soluble aggregates localizing to the neurites and soma of hiPSC-derived neurons. Panels **(A–C)**, respectively, show co-localization of oligomers of K18-WT, K18-V337M, and K18-N279K internalized by SH-SY5Y cells with the FM4-64 endocytosis marker. **(D–F)** Internalized exogenous oligomers of K18-WT, K18-V337M, and K18-N279K, respectively, co-localized with the FM4-64 endocytosis marker in hiPSC-derived neurons. Whilst some of the internalized oligomers of all three protein variants were found in the neurites (arrowheads), others appeared to be present in the soma (arrows). Scale bar = 10 μm for all images.

Previous reports have suggested that K18-WT is internalized by endocytosis (Guo and Lee, 2011; Michel et al., 2014). However, the internalization mechanism of the FTD variants used in this study was not known. The endocytic marker FM4-64^^®^^ (SynaptoRed) was used to stain SH-SY5Y cells during internalization, allowing a preliminary assessment of the localization of the internalized tau. Similar to K18-WT, internalized K18-V337M and K18-N279K oligomers co-localized with FM4-64, suggesting endocytic-dependent internalization (Figure 6A–C). As a control, incubating SH-SY5Y cells seeded with K18 oligomers at 4°C instead of 37°C blocked internalization (Supplementary Figure S9) as shown before for tau K18 and full-length tau 441 (Michel et al., 2014). We next tested if oligomer internalization in a more physiologically relevant cell model, hiPSC-derived neurons, also occurred by endocytosis. Tau taken up by neurons were ostensibly found in both neurites and soma, where they appeared to co-localize with FM4-64 in both compartments (Figure 6D–F). Together, these data suggest that tau K18 oligomers can be internalized by SH-SY5Y cells and hiPSC neurons at least partly through endocytosis.

### Internalized Tau K18 Oligomers Co-localize With Endogenous Tau and the Nuclear Protein Nucleolin

**FIGURE 7:**
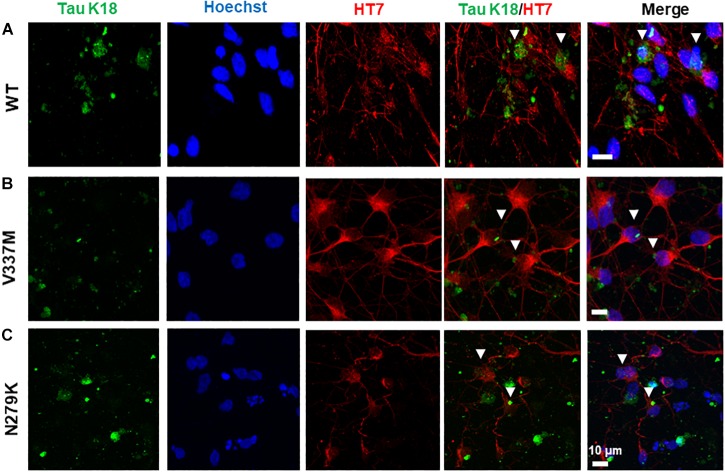
Internalized tau K18 oligomers in hiPSC-derived neurons co-localize with endogenous tau bearing the HT7 (^159^PPGQK^163^) mid-region epitope. Extracellularly applied oligomers of tau K18-WT **(A)**, K18-V337M **(B)**, and K18-N279K **(C)** oligomers internalized in hiPSC-derived neurons co-localized with endogenous tau in the soma and the neurites. White arrowheads show sites of co-localization. Scale bar = 10 μm for all images. Figure extension in Supplementary Figures S8A–C.

The ability of internalized tau oligomers to interact with endogenous tau is a critical step in the inter-cellular propagation of tau (Lasagna-Reeves et al., 2012a; Michel et al., 2014; Usenovic et al., 2015). It is, however, unknown if tau variants of different conformations will interact with endogenous tau differently upon internalization. Internalized K18 oligomers were observed to co-localize with endogenous tau in hiPSC-derived neurons bearing the ^159^PPGQK^163^ epitope (HT7 antibody). All three tau K18 variants co-localized with endogenous tau, occurring both in the neurites and the soma (Figure 7), suggestive of an interaction of internalized tau with endogenous tau.

**FIGURE 8:**
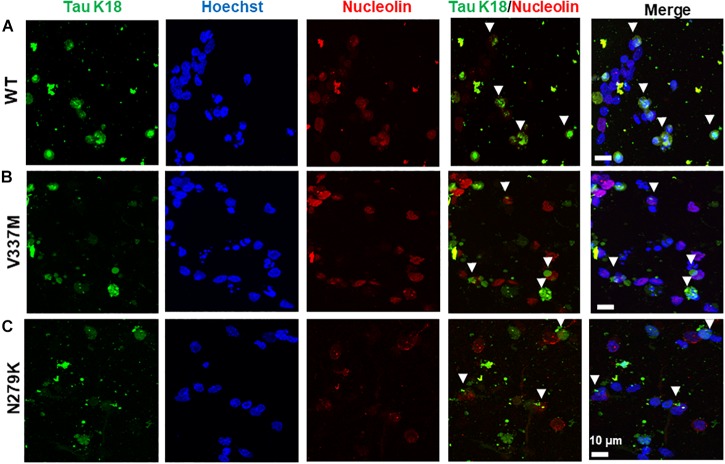
Exogenous tau K18 oligomers internalized in the neurites of hiPSC-derived neurons co-localize with the nucleolar protein nucleolin. Panels **(A–C)** show the co-localization of internalized oligomers of WT, V337M, and N279K tau K18, respectively, with nucleolin. Scale bar = 10 μm for all images. Figure extension in Supplementary Figures S8D–F.

The nuclear localization of both endogenous tau and tau overexpressed from genetic constructs has been reported in both the human and mouse brains (Rady et al., 1995; Liu and Götz, 2013) and cell culture models (Lu et al., 2014; Ritter et al., 2018). Nuclear tau staining is often predominantly apparent in the nucleolus, with tau signals co-localized with those of the nucleolar protein nucleolin (Sjöberg et al., 2006), although more diffuse nuclear staining for endogenous tau has been reported in response to cell stress (Sultan et al., 2011). In SH-SY5Y cells, the internalized tau was not generally detected in the nucleus. Although some association with nucleolin was observed, this was expected to result from the redistribution of the nucleolin protein during the different phases of the cell cycle in these dividing cells (data not shown). Cytoplasmic accumulation of internalized tau, as has been previously demonstrated, was recorded for both cell types. However, in contrast to the SH-SY5Y cells, tau-nucleolin co-localization was observed in hiPSC-derived neurons with nucleolar inclusion of internalized tau for both mutant and WT tau-K18 (Figure 8).

### Internalized Tau K18 Oligomers Do Not Induce LDH-Dependent Cytotoxicity in SH-SY5Y Cells

**FIGURE 9:**
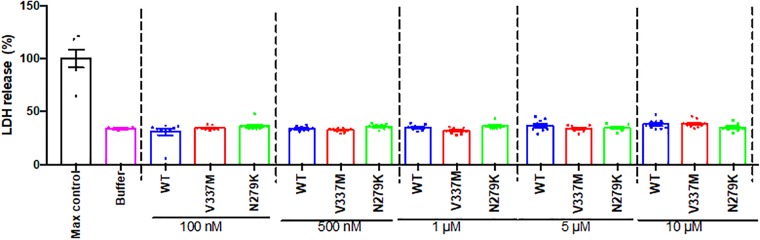
Internalized tau K18 oligomers do not induce LDH-dependent cytotoxicity in SH-SY5Y cells. Increasing concentrations of oligomeric tau K18 variants were applied to SH-SY5Y cells for 72 h, and the cells analyzed for LDH release. We found no significant difference in LDH release between the controls (buffer only or maximum control) and the tau K18 variants at the concentrations studied (*n* = 3 each).

Several studies have shown that internalized tau oligomers impair cell viability and/or induce cell death (Lasagna-Reeves et al., 2010; Guerrero-Muñoz et al., 2015) but others have reported the opposite (Kumar et al., 2014; Kaniyappan et al., 2017). However, the cytotoxicity potential of tau FTD variants is unknown. SH-SY5Y cells were therefore, treated with the respective oligomers and cytotoxicity studied by measuring LDH release. No significant differences in viability were observed between increasing concentrations of WT or mutant K18 oligomers (Figure 9), suggesting that the oligomers did not induce LDH-dependent cytotoxicity over 72 h.

## Discussion

Tau has important functions in the pathogenesis of AD and FTD (Ghetti et al., 2015; Wang and Mandelkow, 2016; Guo et al., 2017). Whilst several theories have been proposed toward understanding disease mechanisms, recent studies have pointed to possible involvement of an inter-neuronal transmission mechanism. Although this hypothesis has strong evidential support (Mohamed et al., 2013; Guo and Lee, 2014; Hu et al., 2016), it remains to be shown why tau isolates from different post-mortem tauopathy brains have distinct internalization and transmission capabilities. In this study, an integrated structural and cell biological approach was used to understand potential links between protein aggregation, conformation and cellular internalization. It was hypothesized that specific disease-associated *MAPT* mutations implicated in familial FTD will alter the structural features (conformation and aggregation) and cellular internalization of WT tau K18. It has been demonstrated that the N279K and V337M FTD-linked mutations do alter tau K18 aggregation and conformation. Moreover, the mutants had enhanced cellular internalization, yet all protein variants were internalized by endocytosis and had shared the same sub-cellular accumulation patterns. These data suggest that structural distinctions between tau variants may influence their cellular internalization properties, with potential implications for the molecular pathogenesis of tauopathies.

Previous studies of the V337M and N279K mutations did not identify any discernible effects on tau aggregation propensity (Barghorn et al., 2000; Combs and Gamblin, 2012). This may have been due to the use of thioflavin fluorescent reporter assays to quantify aggregation (Barghorn et al., 2000; Combs and Gamblin, 2012). Although commonly used to detect cross β-sheet-dependent aggregation (Lindberg et al., 2015), a major pitfall of thioflavin assays is that they appear to be probes of protein conformation instead of aggregation status since different folding behaviors can change the availability of different proteinopathic proteins to the binding sites of the dye (Coelho-Cerqueira et al., 2014; Wong et al., 2016; Hatami et al., 2017). For this reason, the ability of thioflavin dyes to distinguish between conformations of variants of the same protein can sometimes be complicated (Hatami et al., 2017). Although kinetics may remain apparently unaffected, our data using TEM to characterize aggregates suggest that both mutations impact upon the aggregation of K18, a finding supported by atomic force microscopy analysis (data not shown). Immuno-reactivity data to probe conformation supported these findings. These complementary findings in multiple assays build a strong argument in support of the hypothesis that the mutations alter the aggregation and conformation of tau K18. Interestingly, similar profiles were observed for aggregation reactions carried out in the absence of the heparin aggregation inducer. Moreover, the FTD mutations altered the immuno-reactivity of aggregated K18-WT. K18-V337M had lower reactivity to both tau antibodies whilst the N279K mutation almost abolished reactivity to the oligomer-preferring T22 antibody but its reactivity to A0024 was comparable to that of K18-WT. Our results therefore, imply that each mutation shifts protein conformation which in turn dictates the pathway toward forming distinct aggregates. The consistent observation that each protein variant had distinct aggregate structures in several independent assays (both heparin-induced and native-state aggregation) additionally supports mutation-induced differences in protein aggregation and conformations, similar to that previously described for amyloid beta (Hatami et al., 2017). Two main reasons could account for the distinct T22 reactivity even at 0 h, where the samples were monomeric. Firstly, the T22 antibody is thought to selectively recognize an oligomeric conformational epitope. However, the antibody can also react with monomers which presumably share this same epitope (Lasagna-Reeves et al., 2012b). Secondly, the differential T22 binding to the tau K18 variants could also be due to the introduced mutations since the exact epitope of the antibody is unknown (it binds to the repeat domain region – amino acids 244–372 of full length tau 441). It is conceivable that the distinct aggregation and conformational dynamics of the protein variants would also lead to notable differences in epitope availability/recognition. Together, the data provided demonstrate that the V337M and N279K disease-associated mutations induce structural and conformational changes in tau K18 that are evident at distinct stages of aggregation, including oligomers and fibrils.

Tau oligomers are a potential sensitive biomarker for neurodegeneration (Sengupta et al., 2017) and can induce endogenous tau aggregation upon cellular internalization (Lasagna-Reeves et al., 2012a; Michel et al., 2014). Effects of the disease mutations on the cellular uptake of structurally characterized oligomers of tau K18 were therefore investigated. Differences in oligomer uptake were observed in respect to tau mutants. Irrespective of mutation, the uptake of K18 appeared to be endocytic. Uptake of all K18 forms tested highlight that different tau aggregates may each propagate disease by spreading into non-diseased cells. However, differences potentially mediated by aggregate conformation may provide critical insights into shared and distinct molecular mechanisms of tauopathies involving FTD and WT tau.

Consistent with previous reports that used recombinant or brain isolates of tau (Frost et al., 2009; Guo and Lee, 2011; Michel et al., 2014; Falcon et al., 2015; Ando et al., 2016; Calafate et al., 2016), the K18-WT oligomers were most likely internalized by endocytosis in both cell types, with same observed for the FTD variants. Whilst all protein variants appeared to be internalized by endocytosis, co-localized with endogenous tau and were found both in the cytoplasm and nucleus, the degree of internalization differed between the protein variants. We speculative that the new aggregation and/or conformation dynamics recorded in the presence of the FTD mutations may change tau K18 interaction with cell surface proteins that regulate endocytosis, resulting in altered cellular trafficking. In agreement, binding to heparan sulfate proteoglycans regulates the cellular internalization and inter-cellular propagation of aggregated tau, and that blocking the activity or expression of heparan sulfate proteoglycan prevents internalization (Holmes et al., 2013; Rauch et al., 2018). However, it is unknown if WT and mutant tau proteins bind heparan sulfate proteoglycans with the same or different affinities. Furthermore, dysfunctions in key steps in clathrin-mediated endocytosis have been implicated in tau propagation and inclusion formation in AD, FTD and other tauopathies (Ando et al., 2016; Calafate et al., 2016). It remains to be studied if mutation-induced conformational changes do modify tau internalization, propagation and toxicity in an *in vivo* model. However, with the *in vitro* evidence presented in the current study, we can speculate that the altered conformations of tau resulting from the FTD mutations leads to specific changes in the protein’s vesicular uptake or receptor binding, resulting in altered uptake efficiency. Another possibility is that the FTD mutations alter the efficiency of degradation of internalized tau K18 in each cell line, resulting in differential amounts of internalized proteins. It is worth investigating if oligomers of WT and mutated tau are degraded through the same or different pathways, and if the mutations affect clearance efficiency. Previous studies have shown that internalized WT tau oligomers/aggregates and the internalized-endogenous tau aggregate complexes they form intracellularly may be cleared by autophagy (Wu et al., 2013; Usenovic et al., 2015). In agreement, treating hiPSC-derived neurons with rapamycin reduces the amount of internalized tau oligomers-endogenous tau aggregate complexes, suggesting that lysosomal elimination is a common degradation route for tau aggregates.

The ability of internalized exogenous tau aggregates to interact with endogenous tau is critical for the efficient inter-cellular propagation of tau aggregates and the associated neurotoxicity (Lasagna-Reeves et al., 2012a; Michel et al., 2014; Wegmann et al., 2015). The observed co-localization of all three protein variants with endogenous tau suggests that internalized tau K18 and the FTD variants can interact with endogenous tau, indicating the potential for propagation by inducing endogenous tau aggregation, possibly following a templated misfolding mechanism as described previously for tau K18 (Guo and Lee, 2011; Michel et al., 2014). As both the FTD mutants and WT K18 showed similar cellular distribution and localization, it would suggest the potential for prion-like propagation of the FTD mutant tau.

In hiPSC-derived neurons, the co-localization with nucleolin suggests that internalized tau can be taken up into the nucleus, and raises the possibility that internalized tau that gets into the nucleus may have functions in protein biosynthesis. Nuclear tau associates with nucleolin in AD brain, where reduced nuclear tau levels correlated with decreased amounts of nucleolin (Hernández-Ortega et al., 2016). Interaction of aggregated tau, both WT and disease-associated mutated variants, with specific ribosomal proteins can impair the protein synthesis machinery (Meier et al., 2016). Given that pathological tau can impair nucleocytoplasmic transport in AD brains and tau transgenic mice (Eftekharzadeh et al., 2018), and the overexpression of the N279K tau variant in a cell model is associated with increased nuclear localization (Ritter et al., 2018), the effect of tau on the protein synthesis machinery deserves further investigation.

Our findings that tau K18 oligomers, even at high concentrations, did not significantly induce LDH release was unexpected since some previous reports pointed to the contrary (Lasagna-Reeves et al., 2010, 2012a). Nonetheless, our present data is supported by other studies (Kumar et al., 2014; Tepper et al., 2014; Kaniyappan et al., 2017). The origin of these divergent data is unknown but could be explained by differences in oligomer concentrations, preparation methods and cytotoxicity assays. For example, 0.1–1 μM of full length tau-441 oligomers produced by seeding with oligomers from AD brain, amyloid β or α-synuclein were toxic to cells, as measured by the 3-(4,5-dimethylthiazol-2-yl)-5-(3-carboxymethoxyphenyl)-2-(4 sulfophenyl)-2H-tetrazolium (MTS) assay (Lasagna-Reeves et al., 2010, 2012a). On the other hand, applying 10 μM of tau K18 oligomers (recombinant or derived from Sf9 cells) to mouse primary cortical neurons or SH-SY5Y cells did not result in significant cytotoxicity within 3–72 h, as shown by the LDH or MTT (3-(4,5-dimethylthiazol-2-yl)-2,5-diphenyltetrazolium bromide) assays (Kumar et al., 2014; Tepper et al., 2014). Whether or not exogenous tau oligomers induce overt cytotoxicity, their most immediate toxic effect is likely to be an impairment of the structural and functional integrity of neurons and potentially other cell types. In support, internalized tau oligomers impair neural transmission, cause dendritic spine loss and increases in intracellular calcium levels and reactive oxygen species without evidence of overt changes to cell viability (Kumar et al., 2014; Tepper et al., 2014; Usenovic et al., 2015; Yin et al., 2016; Kaniyappan et al., 2017).

Together, these results demonstrate that specific disease-associated single nucleotide polymorphisms in tau can cause drastic changes in protein conformation and aggregation. These distinctive structural and conformational properties may be crucial determinants of the inter-cellular transmission efficiency of the oligomeric forms of these proteins. These findings may provide a molecular explanation as to why tau extracts obtained from different sources (including human tauopathy brains, tau transgenic mice brains, and recombinant preparations) have distinctive propagation potencies and endogenous tau aggregation abilities in both cellular and animal preclinical tauopathy models. Indeed, aggregated tau from transgenic mice brains and recombinant sources have been shown to have distinct conformations that can influence their seeding capabilities (Falcon et al., 2015). It can be argued that critical post-translational modifications such as phosphorylation and the *in vivo* environment in which this process occurs might have contributed to the conformational differences observed. As the recombinant tau proteins used in the present study were not phosphorylated, it can be said that phosphorylation and sites of phosphorylation *per se* may not be responsible for the conformational, structural and molecular activity differences previously observed for tau aggregates from diverse sources. The differential aggregation of the recombinant mutants reinforces the conclusion that specific amino acid modifications are sufficient to induce structural and functional differences in tau-K18. Such structural and functional differences may then be compounded by the differential phosphorylation of the mutant forms of tau or their differential recruitment of endogenous tau.

Importantly, these findings may help to understand why different tauopathies have distinct progression rates, neuropathological features, inclusion formation and types of inclusion (Delisle et al., 1999; Murrell et al., 1999; Sergeant et al., 2005; Ghetti et al., 2015). Furthermore, these results may pave the way for the development of more sensitive, disease- and mutation-specific platforms for early, more accurate diagnosis of tauopathies. Future studies should aim to ascertain if the findings described here are applicable to tau isolates from tauopathy brains: if their reported activity differences can be explained by structural and conformational distinctions.

## Author Contributions

TK, DN, EH, and KM conceived and co-ordinated the study. TK designed and conducted the experiments, analyzed the data, and wrote the first draft of the manuscript. AG, CC-B, and JC made significant contributions to hiPSC experiments, immunocytochemistry, and confocal microscopy. DN, EH, and KM contributed reagents and materials, made important contributions to data interpretation, and critically revised the initial manuscript draft. All authors discussed and commented on the final manuscript.

## Conflict of Interest Statement

The authors declare that the research was conducted in the absence of any commercial or financial relationships that could be construed as a potential conflict of interest.

## Supplementary Material

FIGURE S1 The FTD mutations cause structural changes in the properties of tau K18 filaments. **(A)** Representative TEM images of K18-WT, showing PHFs and SFs. **(B)** Representative electron micrographs of K18-V337M, which formed amorphous aggregates [examples shown in (i), (ii), and (iii)] and short filaments [(iv) and (v)]. **(C)** Representative electron micrographs of K18-N279K, showing bundled filaments of varying shapes and widths. Scale bar = 100 nm for all images.*n* = 3 independent experiments for each protein. This figure is an extension of Figure 2.

FIGURE S2 Uncropped dot blot data used for immuno-reactivity analysis in Figure 4. **(A)** Dot blot intensity data for K18-WT, K18-V337M, and K18-N279K following total tau A0024 reactivity. **(B)** T22 antibody reactivity profiles of K18-WT, K18-V337M, and K18-N279K. For better visualization, the sites of dot blotting are highlighted with black circles.

FIGURE S3 Dominant aggregate conformers of the tau K18 variants are unaffected by seeded aggregation. Endpoint aggregates of K18 WT, V337M, and N279K from 4 days reactions were used to seed the aggregation of each protein in turn. The resulting product, after a further 4 days of incubation at room temperature, were analyzed by TEM imaging (**A**, unseeded; **B**, seeded with WT; **C**, seeded with V337M seeded; and **D**, seeded with N279K). Scale bars = 100 nm for all images.

FIGURE S4 Structural characterization of fluorescently labeled oligomers of tau K18 and its FTD variants. **(A–C)** Representative TEM characterization of tau K18 oligomers labeled with Alexa Fluor 488 conjugated maleimide demonstrating the presence of granular structures for K18-WT, K18-V337M, and K18-N279K, respectively. Insets refer to higher magnification images. Scale bars for all images = 200 nm and 20 nm for insets. **(D)** Non-denaturing SDS PAGE image of labeled tau K18 oligomers.

FIGURE S5 Uptake of extracellularly applied tau K18 oligomers into SH-SY5Y cells. Seeded cells were supplied with 10 μM tau K18 oligomers and incubated for 24 h at 37°C, 5% CO_2_, following which the spent media were removed and the cells washed with warm 10 mM PBS before new tau-free media was added. Uptake of oligomers was demonstrated using confocal microscopy. Panels **(A–C)** show cellular internalization of K18-WT, K18-V337M, and K18-N279K oligomers, respectively. Scale bar = 10 μm for all images.

FIGURE S6 Internalized oligomers of the tau K18 variants localize to the nuclei and cytoplasm of SH-SY5Y cells. Oligomeric aggregates of tau K18-WT **(A)**, K18-V337M **(B)**, and K18-N279K **(C)** internalized by the SH-SY5Y cells appeared to be distributed in the cytoplasm (open arrow heads) and nucleus (filled arrow heads). Scale bars = 10 μm for all images.

FIGURE S7 Higher resolution images of internalized tau K18 oligomers in SH-SY5Y cells. The images here show AF-maleimide-labeled tau K18 oligomers (green) taken up mainly into the cytoplasm of K18-WT, K18-V337M, and K18-N279K (**A–C**, respectively). Scale bar = 10 μm for all images.

FIGURE S8 Colocalization of tau K18 with endogenous tau and nucleolin at high intensity. These figures show digitally increased intensity of selected regions from the images in Figure 7, 8 to better demonstrate colocalization of tau K18 WT, V337M, and N279K with endogenous tau (**A–C**, respectively) or nucleolin (**D–F**, respectively). For better composite color appreciation, colocalization is shown in yellow - merger of red (endogenous tau or nucleolin) and green (tau K18) channels or white – merger of magenta (endogenous tau or nucleolin) and green (tau K18) channels. Arrows point to regions of colocalization.

FIGURE S9 Incubation in cold conditions blocks the internalization of tau K18 oligomers by SH-SY5Y cells. **(A–C)** Oligomer uptake was significantly reduced for tau K18-WT, K18-V337M, and K18-N279K, respectively when SH-SY5Y cells were incubated at 4°C instead of 37°C (as shown in Figure 6).

## Abbreviations

AD: Alzheimer’s disease
ANOVA: analysis of variance
CBD: corticobasal degeneration
DPBS: Dulbecco’s phosphate buffered saline
EDTA: ethylenediaminetetraacetic acid
FTD: frontotemporal dementia, hiPSC-derived neurons, human induced pluripotent stem cell-derived cortical neurons
LMW: low molecular weight
MT: microtubule
NFT: neurofibrillary tangle
PHF: paired helical filaments
PIPES: piperazine-N,N′-bis(2-ethanesulfonic acid)
SF: straight filament
TCEP: tris(2-carboxyethyl)phosphine
TEM: transmission electron microscopy
WT: wild type
