# Supplementary figures and images for: Distinct Conformations, Aggregation and Cellular Internalization of Different Tau Strains
Source: Front Cell Neurosci. 2019 Jul 9;13:296. doi: 10.3389/fncel.2019.00296 (PMC6629824; doi:10.3389/fncel.2019.00296)

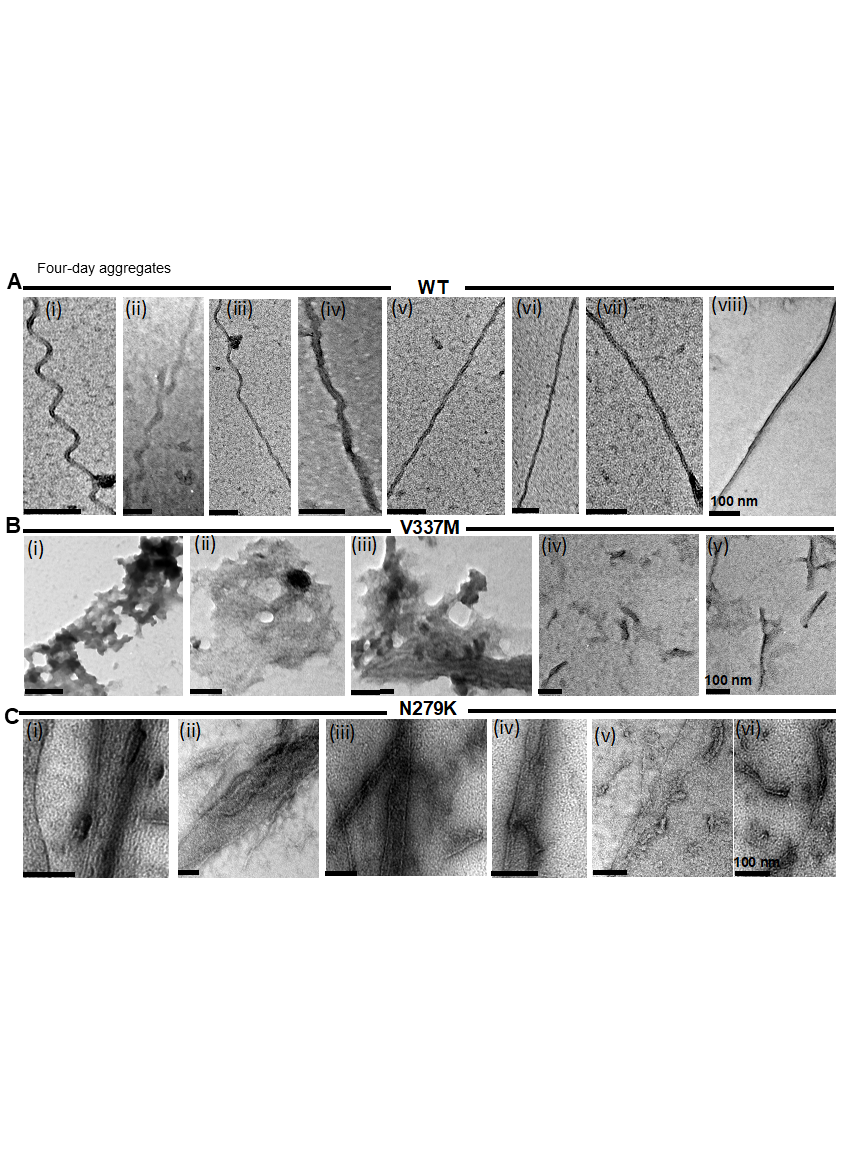

Supplement: FIGURE S1 — The FTD mutations cause structural changes in the properties of tau K18 filaments. (A) Representative TEM images of K18-WT, showing PHFs and SFs. (B) Representative electron micrographs of K18-V337M, which formed amorphous aggregates [examples shown in (i), (ii), and (iii)] and short filaments [(iv) and (v)]. (C) Representative electron micrographs of K18-N279K, showing bundled filaments of varying shapes and widths. Scale bar = 100 nm for all images.n = 3 independent experiments for each protein. This figure is an extension of Figure 2. [file Image_1.TIF]

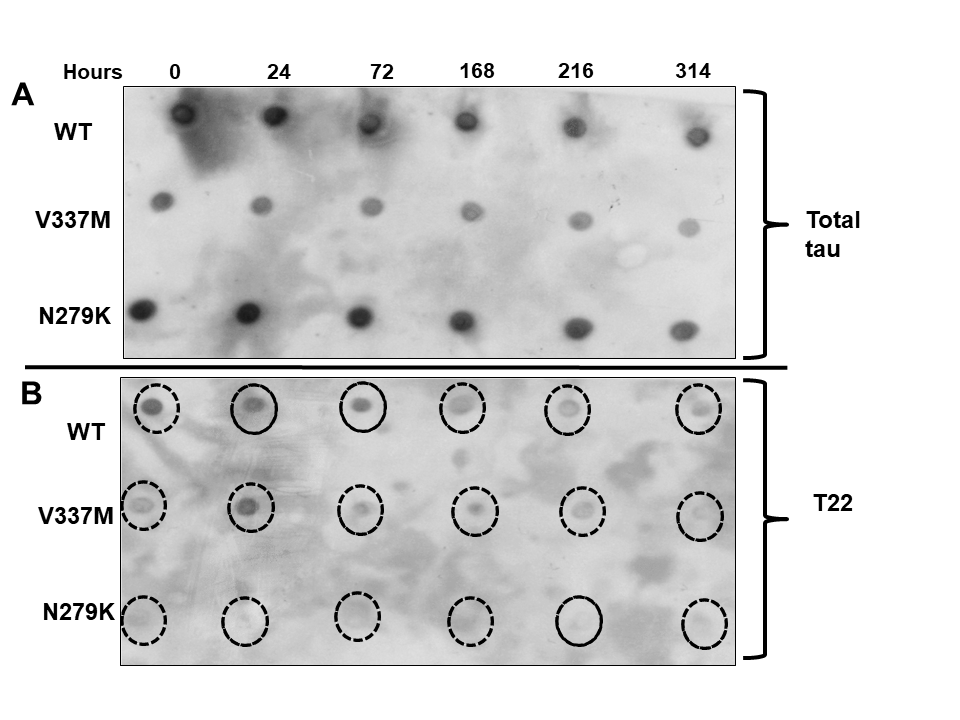

Supplement: FIGURE S2 — Uncropped dot blot data used for immuno-reactivity analysis in Figure 4. (A) Dot blot intensity data for K18-WT, K18-V337M, and K18-N279K following total tau A0024 reactivity. (B) T22 antibody reactivity profiles of K18-WT, K18-V337M, and K18-N279K. For better visualization, the sites of dot blotting are highlighted with black circles. [file Image_2.TIF]

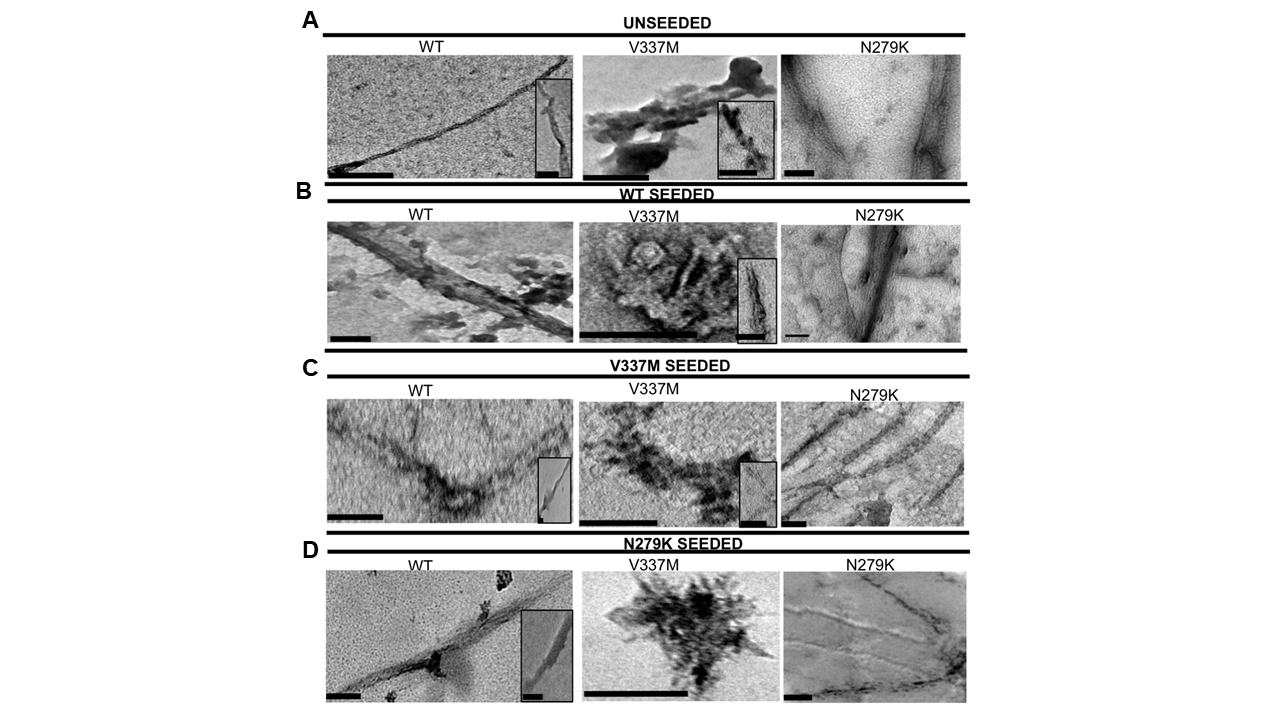

Supplement: FIGURE S3 — Dominant aggregate conformers of the tau K18 variants are unaffected by seeded aggregation. Endpoint aggregates of K18 WT, V337M, and N279K from 4 days reactions were used to seed the aggregation of each protein in turn. The resulting product, after a further 4 days of incubation at room temperature, were analyzed by TEM imaging (A, unseeded; B, seeded with WT; C, seeded with V337M seeded; and D, seeded with N279K). Scale bars = 100 nm for all images. [file Image_3.tif]

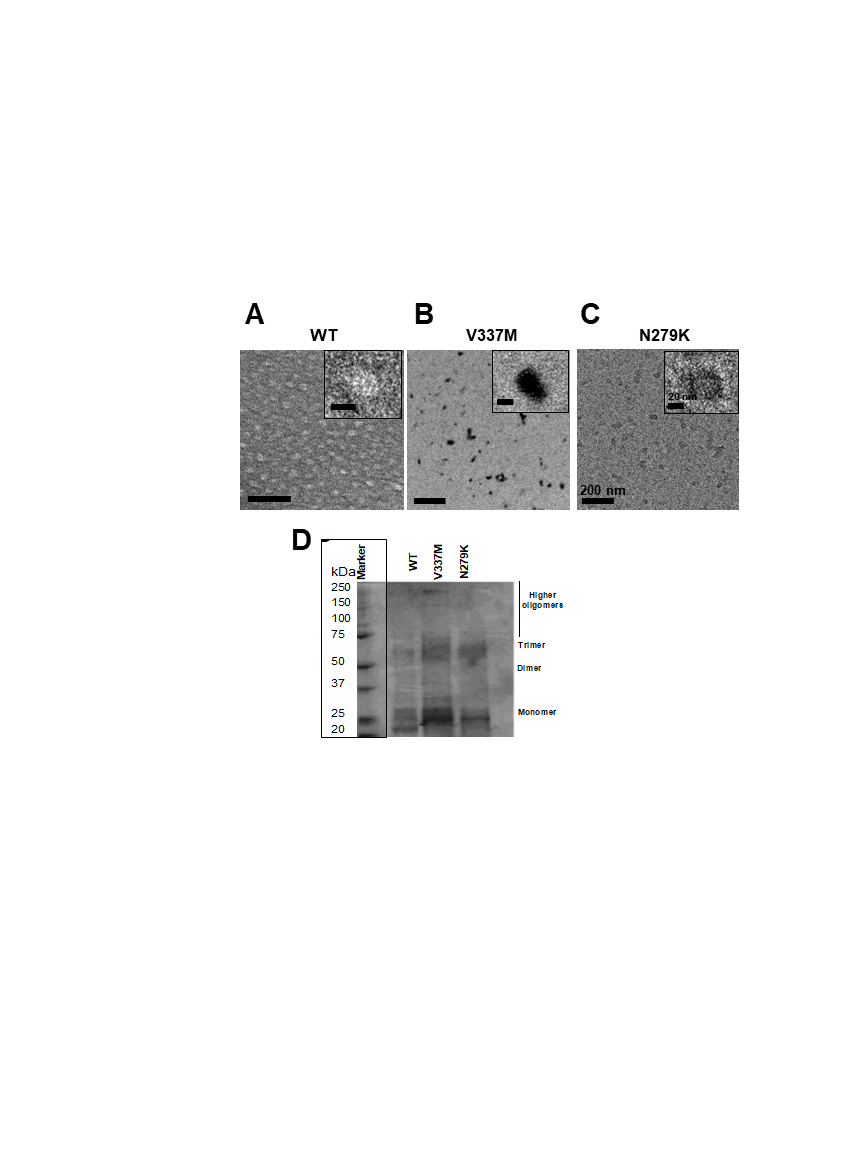

Supplement: FIGURE S4 — Structural characterization of fluorescently labeled oligomers of tau K18 and its FTD variants. (A–C) Representative TEM characterization of tau K18 oligomers labeled with Alexa Fluor 488 conjugated maleimide demonstrating the presence of granular structures for K18-WT, K18-V337M, and K18-N279K, respectively. Insets refer to higher magnification images. Scale bars for all images = 200 nm and 20 nm for insets. (D) Non-denaturing SDS PAGE image of labeled tau K18 oligomers. [file Image_4.TIF]

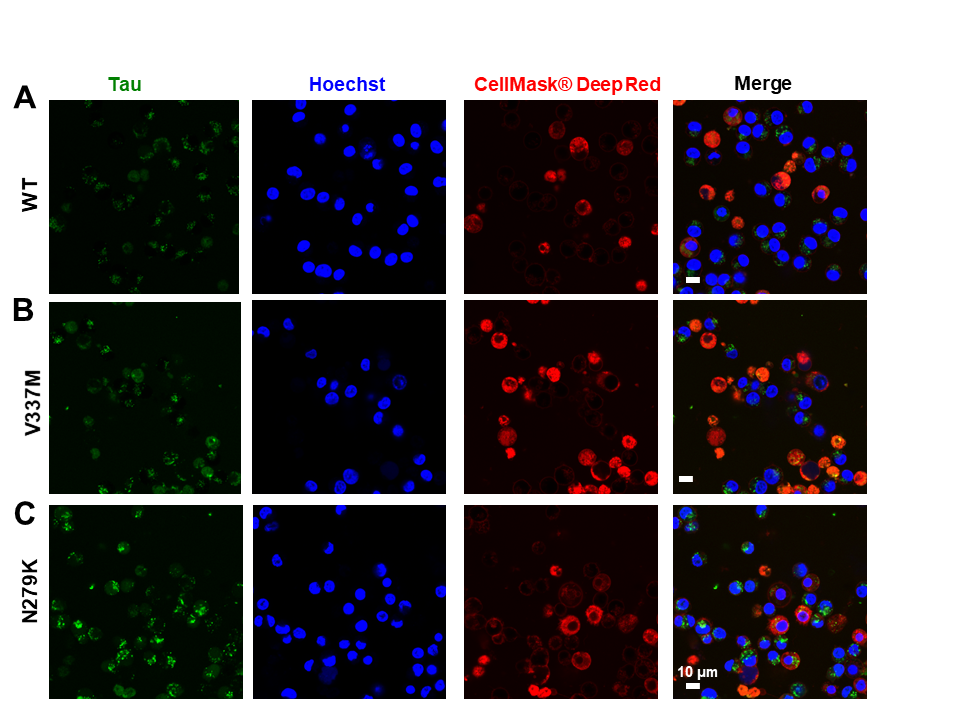

Supplement: FIGURE S5 — Uptake of extracellularly applied tau K18 oligomers into SH-SY5Y cells. Seeded cells were supplied with 10 μM tau K18 oligomers and incubated for 24 h at 37°C, 5% CO2, following which the spent media were removed and the cells washed with warm 10 mM PBS before new tau-free media was added. Uptake of oligomers was demonstrated using confocal microscopy. Panels (A–C) show cellular internalization of K18-WT, K18-V337M, and K18-N279K oligomers, respectively. Scale bar = 10 μm for all images. [file Image_5.TIF]

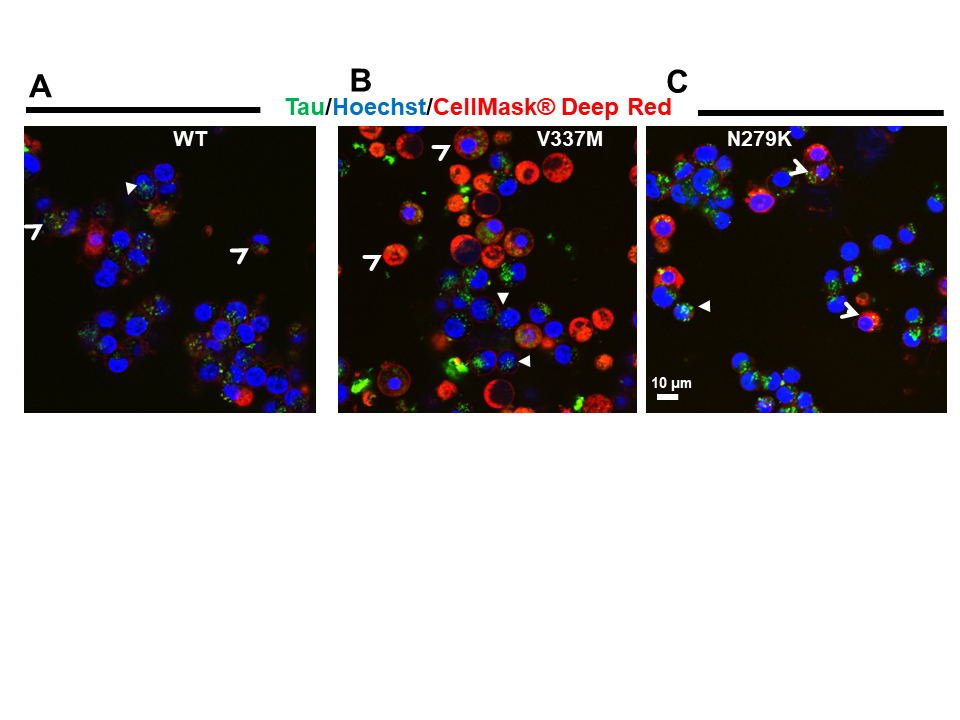

Supplement: FIGURE S6 — Internalized oligomers of the tau K18 variants localize to the nuclei and cytoplasm of SH-SY5Y cells. Oligomeric aggregates of tau K18-WT (A), K18-V337M (B), and K18-N279K (C) internalized by the SH-SY5Y cells appeared to be distributed in the cytoplasm (open arrow heads) and nucleus (filled arrow heads). Scale bars = 10 μm for all images. [file Image_6.TIF]

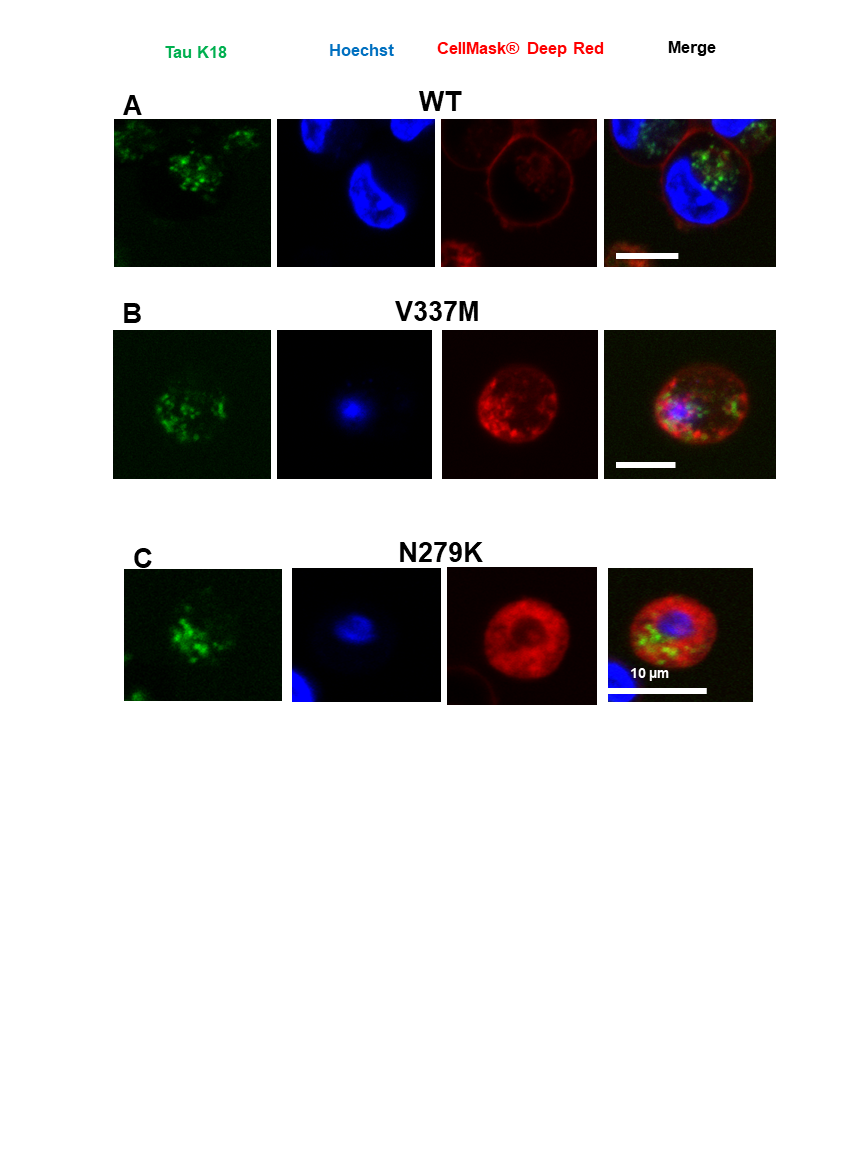

Supplement: FIGURE S7 — Higher resolution images of internalized tau K18 oligomers in SH-SY5Y cells. The images here show AF-maleimide-labeled tau K18 oligomers (green) taken up mainly into the cytoplasm of K18-WT, K18-V337M, and K18-N279K (A–C, respectively). Scale bar = 10 μm for all images. [file Image_7.TIF]

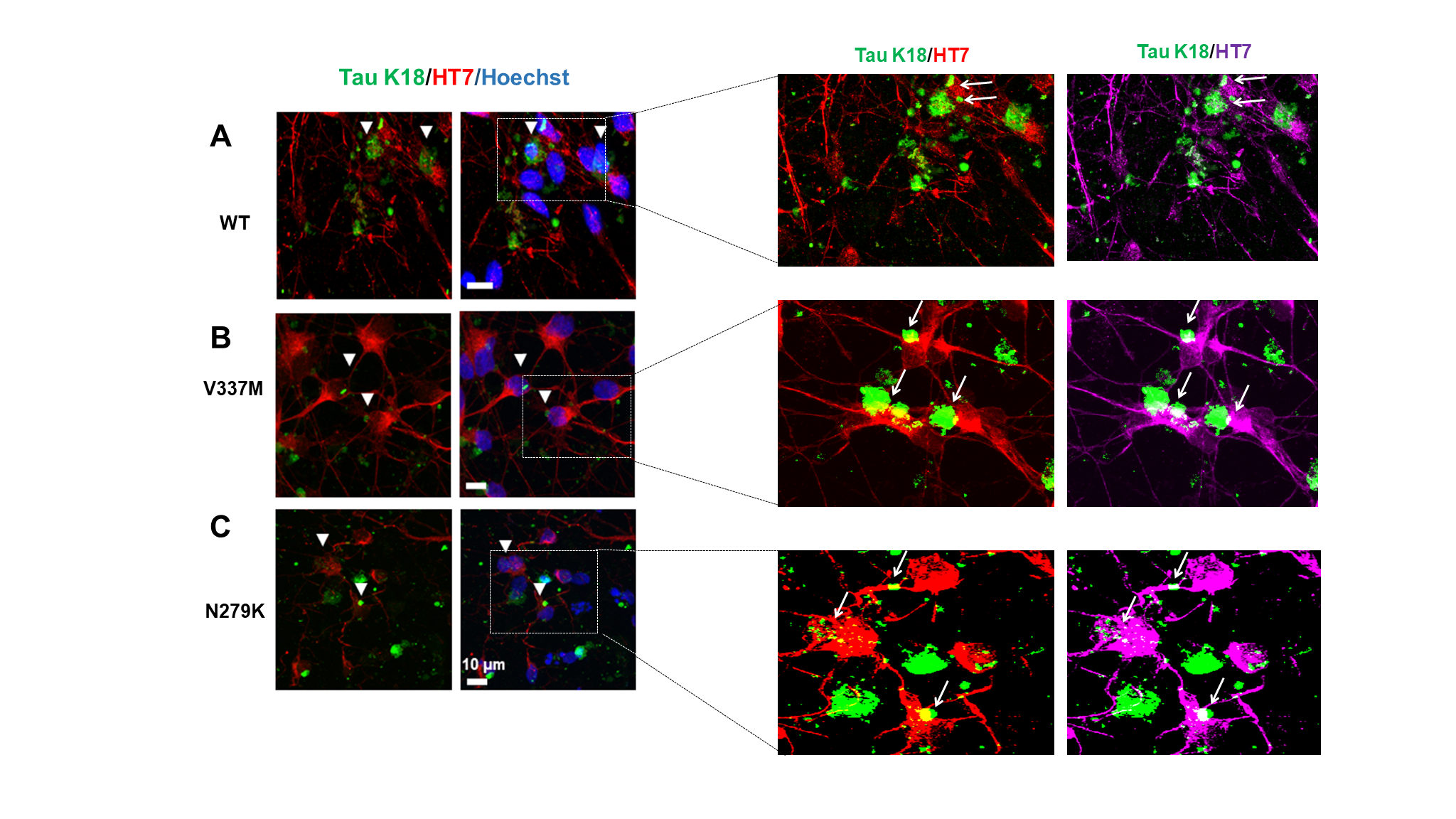

Supplement: FIGURE S8 — Colocalization of tau K18 with endogenous tau and nucleolin at high intensity. These figures show digitally increased intensity of selected regions from the images in Figure 7, 8 to better demonstrate colocalization of tau K18 WT, V337M, and N279K with endogenous tau (A–C, respectively) or nucleolin (D–F, respectively). For better composite color appreciation, colocalization is shown in yellow - merger of red (endogenous tau or nucleolin) and green (tau K18) channels or white – merger of magenta (endogenous tau or nucleolin) and green (tau K18) channels. Arrows point to regions of colocalization. [file Image_8.TIF]

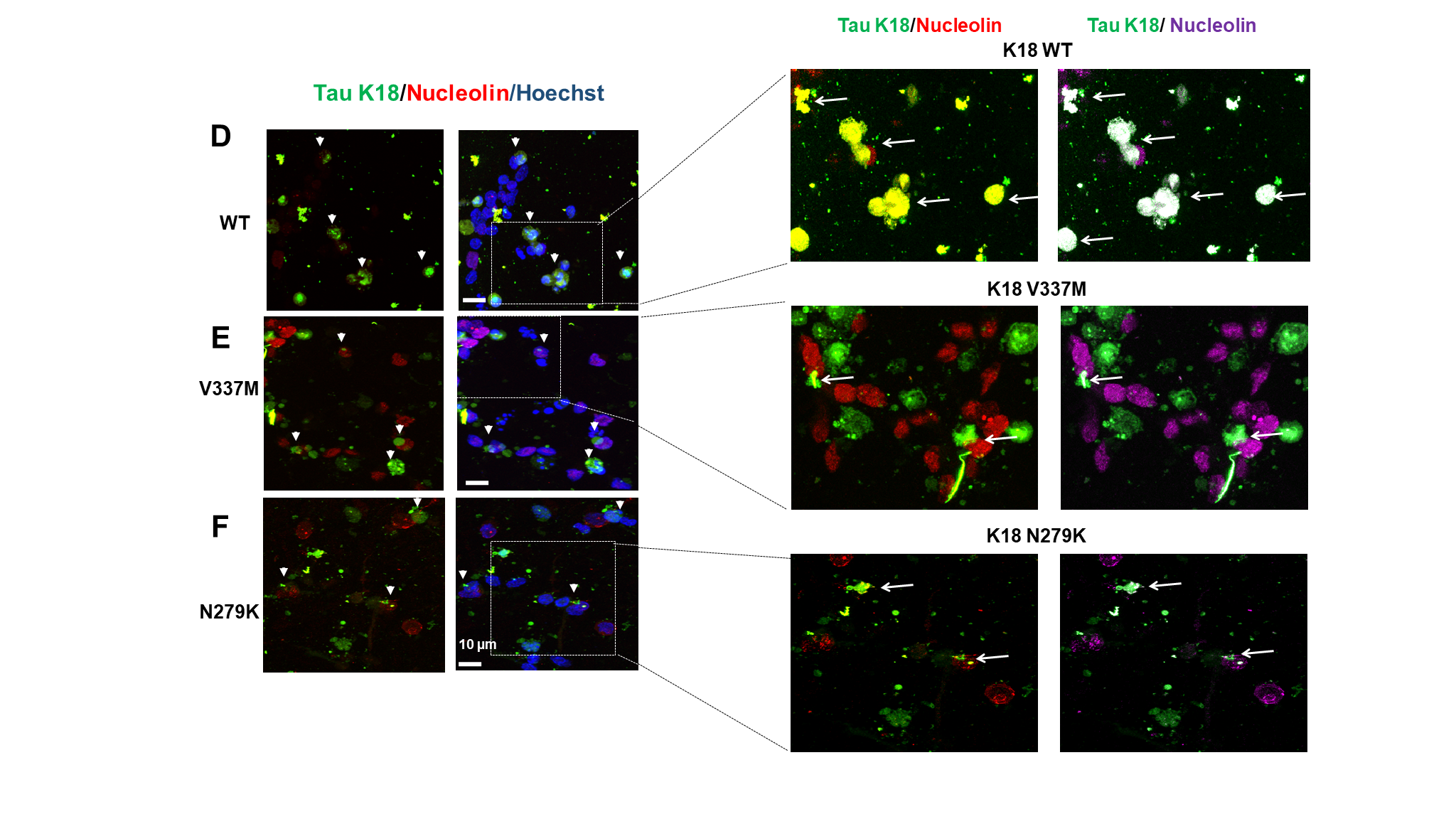

Supplement: FIGURE S9 — Incubation in cold conditions blocks the internalization of tau K18 oligomers by SH-SY5Y cells. (A–C) Oligomer uptake was significantly reduced for tau K18-WT, K18-V337M, and K18-N279K, respectively when SH-SY5Y cells were incubated at 4°C instead of 37°C (as shown in Figure 6). [file Image_9.TIF]

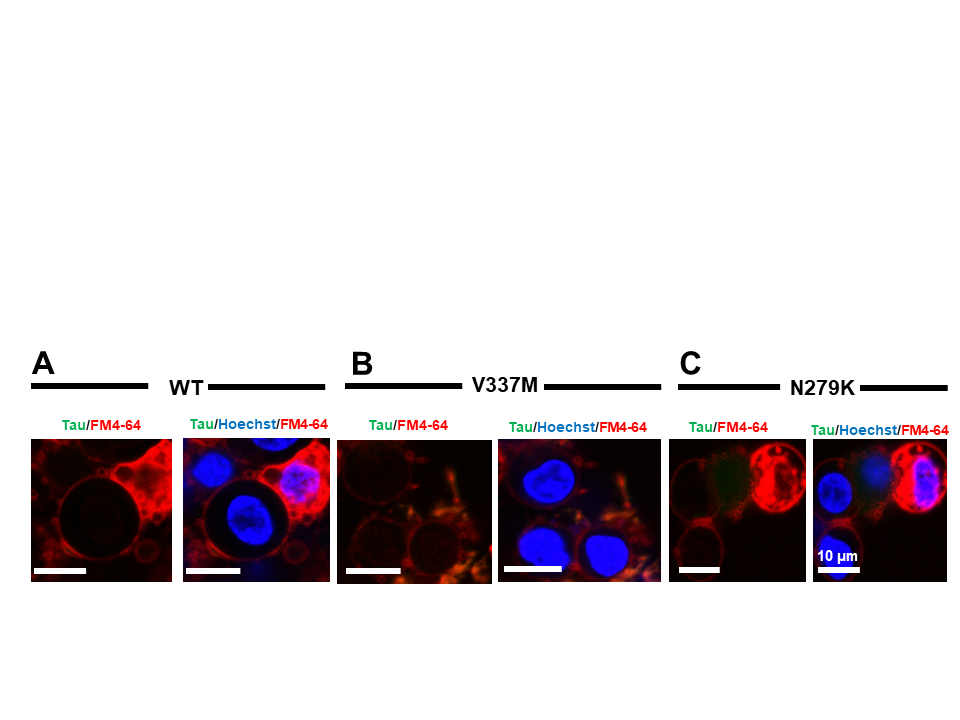

Supplement: Supplementary file 10 [file Image_10.TIF]
